# Supplementary material for: Comparative Compositions of Grain of Bread Wheat, Emmer and Spelt Grown with Different Levels of Nitrogen Fertilisation
Source: Foods. 2023 Feb 16;12(4):843. doi: 10.3390/foods12040843 (PMC9957107; doi:10.3390/foods12040843)
Supplement: Supplementary file 1 [file foods-12-00843-s001.zip › foods-2194484-supplementary.pdf]

Table S1. Means and SEMs of the contents of minerals and metabolites in grains of individual genotypes of the three cereal types grown in four environments.

Some variables required transformation, square root (Sqrt) or log_e (log) to meet the assumptions of the analysis.

	Akteur	Bernstein	Capo	Kometus	RGT Reform	Ramses	Roter Heidfelder	Späths Albjuwel	Attengauer	Bauländer	Comburger	Franckenkorn	Zollernspelt	SEM
%N	1.999	1.973	2.186	1.943	1.883	2.397	2.291	2.409	2.784	2.463	2.618	2.552	2.61	0.04234
log(Ca)	5.802	5.662	6.047	5.805	5.622	5.944	5.732	5.834	5.722	5.641	5.872	5.593	5.869	0.02955
log(Fe)	3.672	3.576	3.608	3.615	3.665	3.673	3.69	3.689	3.985	3.867	3.947	3.996	3.838	0.04708
Mg	1066	1023	1271	1047	1014	1287	1295	1347	1286	1234	1344	1368	1324	17.49
Zn	28.21	25.38	26.91	24.32	24.39	39.62	32.72	38.64	36.69	36.85	38.48	38.28	37.58	0.934
Total phenolics	2887	2751	2914	2851	2761	2562	2881	2915	2863	2723	2937	3011	2934	35.48
raffinose	5.667	5.984	6.18	6.172	5.732	6.805	7.218	6.288	7.017	6.867	6.445	6.883	6.935	0.1033
log(sucrose)	2.269	2.46	2.464	2.415	2.393	2.567	2.653	2.519	2.547	2.56	2.415	2.411	2.416	0.018
log(maltose)	1.499	1.622	1.81	1.593	1.578	2.085	2.219	2.164	1.436	1.475	1.349	1.42	1.355	0.06982
beta galactose	0.3577	0.3705	0.3413	0.3489	0.3413	0.3621	0.3826	0.3697	0.3952	0.3603	0.3729	0.379	0.352	0.00974
Log (total glucose)	0.2976	0.3075	0.3948	0.3156	0.3194	0.4559	0.5059	0.4806	0.3451	0.3284	0.2844	0.284	0.2354	0.0295
fructose	4.794	5.781	4.911	5.495	5.564	3.223	3.977	3.567	4.582	5.799	4.082	4.317	4.348	0.164
glutamine	0.3045	0.2912	0.2896	0.3176	0.3251	0.2655	0.3061	0.2823	0.3126	0.3366	0.3015	0.3123	0.2743	0.01015
glutamate	1.803	1.806	1.868	1.784	1.907	2.084	2.248	2.13	2.17	2.272	2.188	2.299	2.137	0.04053
Log (glycine)	-0.13674	0.02492	0.02179	-0.01742	-0.01497	0.0397	0.11566	-0.00539	0.04826	0.08358	-0.0646	-0.04155	-0.03709	0.0143
Log (alanine)	-2.119	-2.123	-2.066	-2.155	-2.075	-1.969	-1.939	-1.918	-1.945	-1.832	-2.002	-1.934	-1.967	0.01717
aspartate	0.6365	0.6275	0.5903	0.6781	0.7282	0.6785	0.7044	0.6929	0.683	0.8161	0.6269	0.6393	0.7857	0.01778
Log (asparagine)	-0.7219	-0.6903	-0.576	-0.7175	-0.4955	-0.3974	-0.2484	-0.3522	-0.2876	-0.0668	-0.3606	-0.3445	-0.2021	0.03892
GABA	0.2377	0.2329	0.2406	0.233	0.2493	0.2613	0.2833	0.269	0.2785	0.2735	0.2832	0.299	0.277	0.005901
isoleucine	0.08145	0.08108	0.08408	0.08359	0.08678	0.08157	0.09195	0.0868	0.09093	0.09157	0.09245	0.10133	0.08807	0.001474

leucine	0.1214	0.1187	0.1213	0.1169	0.1275	0.1304	0.1443	0.1388	0.1399	0.1432	0.1394	0.1502	0.1352	0.002183
valine	0.1384	0.1368	0.1395	0.1383	0.1465	0.1466	0.1662	0.158	0.1567	0.1676	0.156	0.1693	0.1535	0.00265
threonine	0.1252	0.1172	0.1262	0.1127	0.1342	0.1078	0.1156	0.1111	0.1388	0.1344	0.1354	0.1464	0.1316	0.002963
tryptophan	0.7982	0.5733	0.6684	0.7659	0.6113	0.54	0.7633	0.7014	0.6156	0.6355	0.6701	0.7474	0.7288	0.02068
phenylalanine	0.05856	0.05925	0.0542	0.06088	0.05411	0.06485	0.06858	0.07128	0.06544	0.06907	0.0622	0.06437	0.06326	0.001839
Sqrt (tyrosine)	0.2619	0.2669	0.2529	0.2607	0.258	0.2828	0.2995	0.2912	0.2707	0.2846	0.2673	0.2721	0.2714	0.003785
malic acid	1.729	1.89	1.634	1.854	2.102	2.232	1.997	2.028	1.94	2.307	1.87	1.957	1.955	0.04014
Sqrt (glycine betaine)	1.146	1.159	1.022	1.25	1.143	1.242	1.223	1.183	1.237	1.22	1.291	1.25	1.27	0.009285
choline	0.1687	0.1759	0.2074	0.175	0.1736	0.2102	0.223	0.2201	0.2295	0.2133	0.2142	0.2163	0.2182	0.00288
Sqrt (fumaric acid)	0.07716	0.09289	0.0462	0.07805	0.10661	0.11088	0.07064	0.07964	0.07448	0.09854	0.06113	0.05163	0.0676	0.00308
Log (acetate)	0.1024	0.1071	0.1091	0.1109	0.1156	0.1069	0.1144	0.1124	0.1082	0.1223	0.1091	0.1124	0.1114	0.00329
Sqrt (galactinol)	0.4845	0.4671	0.4658	0.4983	0.4418	0.6403	0.6232	0.5894	0.5122	0.527	0.5242	0.515	0.5299	0.009356
inositol	2.866	3.167	3.137	3.274	2.996	3.137	3.457	2.874	3.644	3.364	3.15	3.243	3.277	0.0491
Log (total amino acids)	1.764	1.749	1.782	1.779	1.815	1.839	1.95	1.883	1.896	1.971	1.87	1.92	1.911	0.01755
Total organic acids	1.908	2.09	1.789	2.043	2.324	2.449	2.182	2.22	2.123	2.528	2.04	2.121	2.134	0.04178
Log (total methyl donors)	0.3916	0.4172	0.2221	0.5516	0.3904	0.5592	0.5399	0.4809	0.5629	0.5286	0.6293	0.5741	0.6031	0.01379
Log (total sugars)	3.268	3.407	3.431	3.387	3.362	3.515	3.612	3.512	3.415	3.479	3.31	3.348	3.343	0.01894

Table S2. Means and SEMs of minerals and metabolites in genotypes of the three cereal types grown at 100 and 200 kg N/Ha.

Some variables required transformation, square root (Sqrt) or $\log_e$ (log) to meet the assumptions of the analysis.

	100 kg N												
	bread wheat					emmer			spelt				
	Akteur	Bernstein	Capo	Kometus	RGT Reform	Ramses	Roter Heidfelder	Späths Albjuwel	Attengauer	Bauländer	Comburger	Franckenkorn	Zollernspelt
%N	1.736	1.72	1.94	1.728	1.696	2.187	1.982	2.118	2.541	2.206	2.342	2.269	2.411
log(Ca)	5.77	5.613	6.004	5.781	5.54	5.938	5.686	5.752	5.718	5.597	5.856	5.579	5.799
log(Fe)	3.617	3.446	3.593	3.594	3.574	3.603	3.606	3.596	4.063	3.778	3.861	3.879	3.738
Mg	1076	1003	1240	1056	1030	1338	1312	1374	1298	1248	1332	1362	1314
Zn	26.66	22.89	24.78	24.07	23.64	38.3	30.81	36.57	33.63	33.9	35.06	35.16	34.12
total phenolics	2828	2689	2902	2750	2761	2500	2821	2904	2943	2686	3009	3054	2976
raffinose	5.652	5.977	6.18	6.226	5.716	7.173	7.6	6.458	7.213	6.851	6.546	6.969	7.002
Log (sucrose)	2.265	2.473	2.48	2.428	2.383	2.608	2.725	2.572	2.574	2.577	2.434	2.42	2.435
Log (maltose)	1.518	1.667	1.842	1.561	1.541	1.997	2.179	2.261	1.511	1.499	1.375	1.378	1.343
galactose	0.3597	0.391	0.3414	0.3479	0.3454	0.3777	0.3625	0.3945	0.414	0.355	0.3801	0.3782	0.3513
Log (total glucose)	0.2817	0.313	0.39	0.2868	0.292	0.4227	0.4773	0.5276	0.387	0.3122	0.2893	0.2675	0.2251
fructose	5.109	6.131	5.208	5.854	5.77	3.483	4.377	3.828	4.814	6.021	4.197	4.517	4.493

glutamine	0.2844	0.263	0.2664	0.2967	0.2878	0.254	0.2871	0.2588	0.291	0.2774	0.2949	0.3147	0.2694
glutamate	1.719	1.846	1.838	1.717	1.859	2.063	2.215	2.111	2.113	2.155	2.149	2.238	2.092
Log (glycine)	-0.1303	0.045	0.04277	-0.0007	-0.0196	0.06939	0.16616	0.02287	0.078	0.09754	-0.0457	-0.0266	-0.0193
Log (alanine)	-2.183	-2.116	-2.091	-2.186	-2.121	-2.014	-1.976	-1.976	-1.976	-1.927	-2.022	-1.943	-1.998
aspartate	0.5911	0.623	0.5732	0.6342	0.6907	0.6665	0.683	0.6376	0.642	0.7901	0.6111	0.6305	0.7537
Log (asparagine)	-0.8322	-0.717	-0.6375	-0.7881	-0.5851	-0.4637	-0.3049	-0.4833	-0.42	-0.1998	-0.4526	-0.4181	-0.3065
GABA	0.2275	0.24	0.2372	0.2251	0.2443	0.261	0.2847	0.2667	0.273	0.2628	0.2785	0.2929	0.2718
isoleucine	0.07688	0.082	0.08283	0.08012	0.08395	0.0818	0.08986	0.08446	0.089	0.08459	0.09099	0.10054	0.08497
leucine	0.1146	0.12	0.1194	0.1124	0.1231	0.1297	0.1406	0.1363	0.137	0.1338	0.1364	0.1469	0.1309
valine	0.1293	0.137	0.1368	0.1324	0.1402	0.1445	0.1587	0.1518	0.152	0.1521	0.1525	0.166	0.1467
threonine	0.1218	0.119	0.1273	0.1116	0.1307	0.1079	0.1167	0.1075	0.137	0.1265	0.1322	0.1473	0.1275
tryptophan	0.7117	0.575	0.6863	0.7095	0.5916	0.5599	0.7389	0.7027	0.675	0.6355	0.7158	0.7866	0.7424
phenylalanine	0.05511	0.061	0.05377	0.05776	0.05274	0.06446	0.06335	0.07214	0.066	0.06446	0.06119	0.06411	0.05907
sqrt(tyrosine)	0.2565	0.271	0.2526	0.2571	0.2552	0.2884	0.3066	0.2959	0.272	0.28	0.2663	0.2693	0.2671
malic acid	1.654	1.947	1.652	1.83	2.075	2.17	1.96	2.013	1.922	2.201	1.822	1.94	1.914
Sqrt (glycine betaine)	1.142	1.168	1.046	1.229	1.141	1.246	1.229	1.186	1.251	1.219	1.318	1.269	1.28
choline	0.1614	0.169	0.2022	0.1718	0.1692	0.2116	0.2199	0.2129	0.225	0.2086	0.2095	0.2116	0.2141
Sqrt (fumeric acid)	0.07257	0.09	0.04427	0.07297	0.09559	0.10798	0.07116	0.08162	0.068	0.09616	0.0584	0.04781	0.06359
Log (acetate)	0.1021	0.113	0.1029	0.1072	0.1099	0.1134	0.1117	0.1106	0.103	0.1147	0.103	0.1079	0.1039
Sqrt (galactinol)	0.5122	0.527	0.5242	0.515	0.5299	0.6504	0.6248	0.6165	0.516	0.5245	0.5192	0.5087	0.5258
inositol	2.817	3.173	3.159	3.27	2.979	3.254	3.557	2.839	3.771	3.395	3.251	3.344	3.344
log(total amino acids)	1.707	1.755	1.771	1.74	1.776	1.833	1.938	1.855	1.872	1.918	1.857	1.909	1.884

total organic acids	1.829	2.15	1.799	2.01	2.28	2.392	2.143	2.205	2.093	2.412	1.983	2.096	2.081
Log (total methyl donors)	0.3802	0.426	0.2571	0.5185	0.3839	0.5663	0.5478	0.4808	0.58	0.5245	0.6625	0.5967	0.6133
Log (sugar)	3.279	3.431	3.456	3.398	3.353	3.525	3.641	3.568	3.453	3.501	3.328	3.349	3.355

	200 kg N													SEM
	bread wheat				emmer			spelt						
	Akteur	Bernstein	Capo	Kometus	RGT Reform	Ramses	Roter Heidfelder	Späths Albjuwel	Attengauer	Bauländer	Comburger	Franckenkorn	Zollernspelt	
%N	2.263	2.226	2.432	2.158	2.071	2.607	2.601	2.7	3.026	2.72	2.894	2.835	2.809	0.06828
log(Ca)	5.833	5.711	6.091	5.829	5.705	5.95	5.779	5.916	5.725	5.685	5.889	5.606	5.94	0.04192
log(Fe)	3.727	3.706	3.623	3.636	3.757	3.742	3.775	3.783	3.906	3.957	4.032	4.114	3.938	0.06632
Mg	1057	1043	1301	1038	997	1235	1278	1321	1274	1221	1355	1373	1335	26.07
Zn	29.76	27.86	29.04	24.56	25.14	40.94	34.64	40.72	39.76	39.79	41.9	41.4	41.03	1.442
Total phenolics	2946	2813	2925	2952	2761	2623	2940	2925	2783	2760	2866	2968	2893	5254
raffinose	5.681	5.992	6.181	6.117	5.749	6.438	6.837	6.118	6.822	6.883	6.344	6.796	6.869	0.1467
Log (sucrose)	2.273	2.448	2.447	2.403	2.402	2.526	2.581	2.467	2.52	2.544	2.397	2.402	2.398	0.02481
Log (maltose)	1.48	1.577	1.779	1.625	1.615	2.172	2.26	2.068	1.361	1.451	1.322	1.461	1.367	0.09744
galactose	0.3556	0.3501	0.3413	0.3498	0.3372	0.3465	0.403	0.3449	0.3763	0.3657	0.3656	0.3797	0.3526	0.01435
Log (total glucose)	0.3135	0.3018	0.3996	0.3445	0.3468	0.4892	0.535	0.4336	0.303	0.3447	0.2796	0.3005	0.2457	0.04158
fructose	4.48	5.431	4.614	5.135	5.359	2.963	3.578	3.306	4.349	5.577	3.967	4.116	4.202	0.2482
glutamine	0.3245	0.3193	0.3127	0.3384	0.3624	0.2771	0.325	0.3059	0.3345	0.3958	0.3081	0.3099	0.2793	0.01437
glutamate	1.887	1.766	1.898	1.851	1.955	2.105	2.28	2.149	2.228	2.389	2.228	2.36	2.182	0.06024
Log (glycine)	-0.1431	0.00491	0.0008	-0.0342	-0.0103	0.01002	0.065	-0.0337	0.01823	0.06962	-0.0835	-0.0565	-0.0549	0.02011
Log (alanine)	-2.055	-2.13	-2.04	-2.124	-2.029	-1.924	-1.901	-1.86	-1.915	-1.737	-1.982	-1.924	-1.936	0.02597
aspartate	0.6819	0.6321	0.6074	0.7221	0.7656	0.6905	0.726	0.7481	0.7246	0.8422	0.6427	0.6481	0.8177	0.02613
Log (asparagine)	-0.6116	-0.6639	-0.5144	-0.6468	-0.4059	-0.3312	-0.192	-0.2212	-0.1553	0.0661	-0.2687	-0.2709	-0.0977	0.05474
GABA	0.2479	0.2253	0.2441	0.2409	0.2543	0.2616	0.282	0.2712	0.2843	0.2841	0.288	0.305	0.2822	0.008687
isoleucine	0.08602	0.08052	0.08534	0.08706	0.0896	0.08134	0.094	0.08914	0.09306	0.09855	0.09392	0.10212	0.09116	0.002208
leucine	0.1283	0.1177	0.1233	0.1215	0.1318	0.1311	0.148	0.1414	0.143	0.1526	0.1423	0.1536	0.1395	0.003224
valine	0.1475	0.1363	0.1421	0.1442	0.1529	0.1486	0.174	0.1641	0.1619	0.1831	0.1595	0.1726	0.1604	0.003958
threonine	0.1287	0.1153	0.1252	0.1137	0.1376	0.1078	0.114	0.1147	0.1403	0.1422	0.1385	0.1456	0.1356	0.004198

tryptophan	0.8847	0.5719	0.6505	0.8222	0.6311	0.5202	0.788	0.7	0.5564	0.6356	0.6245	0.7082	0.7152	0.02951
phenylalanine	0.062	0.05778	0.05462	0.064	0.05549	0.06524	0.074	0.07042	0.06443	0.07368	0.06321	0.06462	0.06745	0.002636
Sqrt (tyrosine)	0.2673	0.2624	0.2531	0.2643	0.2608	0.2771	0.292	0.2864	0.2695	0.2891	0.2682	0.2749	0.2757	0.005324
malic acid	1.803	1.833	1.616	1.879	2.129	2.293	2.034	2.043	1.958	2.413	1.918	1.973	1.997	0.06305
Sqrt (glycine betaine)	1.15	1.151	0.998	1.272	1.145	1.237	1.216	1.18	1.223	1.22	1.264	1.231	1.26	0.01613
choline	0.176	0.1824	0.2125	0.1781	0.1779	0.2087	0.226	0.2272	0.2342	0.2179	0.2189	0.2211	0.2223	0.004091
Sqrt (fumeric acid)	0.08176	0.0954	0.04812	0.08314	0.11763	0.11377	0.07	0.07767	0.08057	0.10093	0.06385	0.05546	0.0716	0.00507
Log (acetate)	0.1027	0.1009	0.1153	0.1145	0.1213	0.1005	0.117	0.1141	0.1136	0.1298	0.1153	0.1168	0.1189	0.004889
Sqrt (galactinol)	0.4859	0.4629	0.4714	0.4997	0.4375	0.6302	0.622	0.5623	0.5085	0.5295	0.5292	0.5213	0.5339	0.01304
inositol	2.914	3.162	3.115	3.278	3.014	3.021	3.358	2.909	3.516	3.334	3.049	3.143	3.211	0.06833
Log(total amino acids)	1.82	1.743	1.794	1.818	1.854	1.845	1.962	1.91	1.919	2.023	1.883	1.931	1.938	0.02526
Total organic acids	1.988	2.029	1.779	2.076	2.368	2.507	2.221	2.235	2.153	2.644	2.097	2.145	2.187	0.06654
Log (total methyl donors)	0.4031	0.4086	0.1872	0.5848	0.3969	0.5521	0.532	0.4809	0.5462	0.5328	0.5961	0.5515	0.593	0.02359
log(total sugars)	3.258	3.383	3.407	3.376	3.37	3.505	3.584	3.456	3.377	3.457	3.291	3.346	3.332	0.02684

Table S3. Means and SEMs of contents of polar metabolites in grains of the three cereal types grown in four environments at two N levels

Some variables required transformation, square root (Sqrt) or $\log_e$ (log) to meet the assumptions of the analysis.

	100			200				
	bread wheat	emmer	spelt	bread wheat	emmer	spelt	SEMemmer	SEM bread&spelt
Raffinose	5.95	7.077	6.916	5.944	6.465	6.743	0.08549	0.06681
log(sucrose)	2.406	2.635	2.488	2.395	2.525	2.452	0.01354	0.00984
log(maltose)	1.626	2.146	1.421	1.615	2.166	1.392	0.05472	0.041116
galactose	0.3571	0.3783	0.3757	0.3468	0.3647	0.368	0.008904	0.007348
log(total glucose)	0.3127	0.4759	0.2962	0.3412	0.4858	0.2947	0.02384	0.01834
fructose	5.614	3.896	4.809	5.004	3.282	4.442	0.1605	0.1364
glutamine	0.2797	0.2666	0.2894	0.3315	0.3027	0.3255	0.00831	0.006448
glutamate	1.796	2.13	2.149	1.871	2.178	2.277	0.03793	0.03164
log(glycine)	-0.01257	0.08614	0.01684	-0.03639	0.01384	-0.0214	0.011484	0.008795
log(alanine)	-2.139	-1.989	-1.973	-2.076	-1.895	-1.899	0.01678	0.01424
aspartate	0.6224	0.6624	0.6854	0.6818	0.7215	0.735	0.01617	0.01331
log(asparagine)	-0.7119	-0.4173	-0.3594	-0.5685	-0.2481	-0.1453	0.03125	0.02393
GABA	0.2349	0.2708	0.2757	0.2425	0.2716	0.2887	0.005389	0.004445
isoleucine	0.08108	0.08537	0.08998	0.08571	0.08817	0.09576	0.001406	0.001182
leucine	0.1178	0.1355	0.1369	0.1245	0.1401	0.1462	0.00201	0.001665
valine	0.1352	0.1517	0.1538	0.1446	0.1621	0.1675	0.00251	0.002105

threonine	0.1221	0.1107	0.1342	0.1241	0.1123	0.1405	0.002433	0.01892
tryptophan	0.6547	0.6671	0.711	0.7121	0.6693	0.648	0.01735	0.01367
phenylalanine	0.05602	0.06665	0.06306	0.05878	0.06982	0.06668	0.001563	0.001241
sqrt(tyrosine)	0.2586	0.297	0.2709	0.2616	0.2853	0.2755	0.00304	0.002329
malic acid	1.831	2.048	1.96	1.852	2.123	2.052	0.04274	0.03737
sqrt(glycine betaine)	1.145	1.22	1.267	1.143	1.211	1.24	0.01205	0.01105
choline	0.1748	0.2148	0.2137	0.1854	0.2207	0.2229	0.002383	0.001862
sqrt(fumeric acid)	0.07516	0.08692	0.06687	0.08521	0.08719	0.07448	0.003613	0.003245
log(acetate)	0.1071	0.1119	0.1065	0.1109	0.1105	0.1189	0.003078	0.002567
sqrt(galactinol)	0.4716	0.6305	0.5188	0.4715	0.6047	0.5245	0.007351	0.005554
Inositol	3.08	3.216	3.421	3.097	3.096	3.25	0.03813	0.02848

Table S4. p values from ANOVA of the contents of polar metabolites in grains of the three cereal types grown in four environments at two N levels

Statistically significant values (p<0.05) are highlighted.

Some variables required transformation, square root (Sqrt) or $\log_e$ (log) to meet the assumptions of the analysis.

	Nlevel	Grain	Nlevel. Grain	Grain.genotype. Bread	Grain.genotype. Emmer	Grain.genotype. Spelt	Nlevel. Grain. genotype. Bread wheat	Nlevel. Grain. genotype. Emmer	Nlevel.Grain.genotype. Spelt
rtaffinose	0.005	<0.001	<0.001	<0.001	<0.001	0.001	0.988	0.271	0.709
Log (sucrose)	<0.001	<0.001	<0.001	<0.001	<0.001	<0.001	0.798	0.465	0.973
Log (maltose)	0.747	<0.001	0.889	0.029	0.392	0.662	0.883	0.155	0.811
galactose	0.225	0.002	0.918	0.17	0.327	0.02	0.518	0.003	0.431
Log (total glucose)	0.435	<0.001	0.721	0.143	0.489	0.078	0.909	0.099	0.593
fructose	0.007	<0.001	0.423	<0.001	0.006	<0.001	0.967	0.787	0.984
glutamine	<0.001	0.005	0.399	0.05	0.019	<0.001	0.743	0.7	<0.001
glutamate	0.025	<0.001	0.361	0.166	0.014	0.022	0.231	0.969	0.677
Log (glycine)	<0.001	<0.001	0.068	<0.001	<0.001	<0.001	0.676	0.469	0.937
Log (alanine)	<0.001	<0.001	0.492	0.001	0.111	<0.001	0.053	0.714	0.005
aspartate	0.002	<0.001	0.893	<0.001	0.589	<0.001	0.395	0.196	0.713
Log (asparagine)	<0.001	<0.001	0.352	<0.001	0.022	<0.001	0.624	0.34	0.78

GABA	0.114	<0.001	0.366	0.263	0.029	0.024	0.25	0.912	0.957
isoleucine	0.005	<0.001	0.383	0.048	<0.001	<0.001	0.12	0.399	0.029
leucine	0.002	<0.001	0.331	0.011	<0.001	<0.001	0.112	0.623	0.183
valine	<0.001	<0.001	0.415	0.081	<0.001	<0.001	0.108	0.329	0.007
threonine	0.058	<0.001	0.417	<0.001	0.181	0.006	0.592	0.501	0.317
tryptophan	0.898	0.596	<0.001	<0.001	<0.001	<0.001	0.002	0.317	0.244
phenylalanine	0.022	<0.001	0.933	0.026	0.048	0.086	0.307	0.049	0.127
Sqrt (tyrosine)	0.908	<0.001	0.008	0.118	0.008	0.018	0.399	0.909	0.803
malic acid	0.202	<0.001	0.345	<0.001	<0.001	<0.001	0.191	0.722	0.511
Sqrt (glycine betaine)	0.358	<0.001	0.087	<0.001	<0.001	<0.001	0.014	0.951	0.305
Choline	<0.001	<0.001	0.535	<0.001	0.005	<0.001	0.852	0.109	1
Sqrt (fumeric acid)	0.121	<0.001	0.083	<0.001	<0.001	<0.001	0.407	0.625	0.896
Log (acetate)	0.021	0.027	0.008	<0.001	0.326	0.018	0.029	0.219	0.752
Sqrt (galactinol)	0.399	<0.001	0.061	<0.001	<0.001	0.605	0.935	0.146	0.951
inositol	0.002	<0.001	0.009	<0.001	<0.001	<0.001	0.883	0.06	0.678

Table S5. Means and SEMs for AXOS and GOS in grains of the three cereal types grown at 100 and 200 kgN/Ha

Some variables required transformation, square root (Sqrt) or $\log_e$ (log) to meet the assumptions of the analysis.

	100			200				
	bread	emmer	spelt	bread	emmer	spelt	SEM emmer	SEM bread&spelt
sqrt(Xylose)	2.761	2.3	2.753	2.803	2.318	2.736	0.03207	0.02603
sqrt(Xyl2)	3.08	2.743	3.105	3.131	2.745	3.15	0.03744	0.03075
sqrt(Xyl3)	1.272	0.979	1.226	1.287	0.921	1.206	0.03594	0.02818
sqrt(Xyl5)	0.8531	0.9449	0.9822	0.825	0.9258	0.9673	0.03181	0.02469
sqrt(G3)	2.41	2.014	2.537	2.326	1.919	2.431	0.02493	0.02059
sqrt(G4)	1.547	1.264	1.687	1.509	1.22	1.623	0.01569	0.0125
sqrt(XA3XX)	2.063	1.541	1.872	2.06	1.513	1.843	0.01834	0.01468
sqrt(XA2_3XX)	1.58	1.196	1.354	1.586	1.171	1.344	0.0143	0.01082
sqrt(XA3A3XX)	1.0305	0.6737	0.8593	1.007	0.6399	0.8471	0.01388	0.01106
sqrt(XA3XA3XX)	0.4605	0.3669	0.4009	0.4688	0.3601	0.4037	0.006016	0.004579
sqrt(XA3A2_3XX)	0.7378	0.5891	0.6429	0.7334	0.5723	0.663	0.011673	0.008902
sqrt(XA3XA2_3XX)	1.0098	0.831	0.9662	1.0092	0.8165	0.9423	0.01261	0.00976
$\log_e$ (TOT-AX)	3.369	3.004	3.308	3.388	2.99	3.306	0.01884	0.01552
$\log_e$ (TOT-BG)	2.097	1.726	2.225	2.032	1.637	2.143	0.02135	0.01754
Ratio G3:G4 GOS	2.429	2.54	2.268	2.381	2.477	2.252	0.02194	0.01725
Ratio TOT-AXOS: TOT-BG	3.596	3.619	2.968	3.915	3.895	3.212	0.05261	0.04096
Sqrt (Ratio M:D AXOS)	1.436	1.366	1.447	1.425	1.36	1.439	0.00587	0.004698

	100+200 bread	100+200 emmer	100+200 spelt	SEM emmer	SEM bread&spelt
sqrt(Xylose)	2.782	2.309	2.745	0.02095	0.01622
sqrt(Xyl2)	3.106	2.744	3.128	0.02389	0.01851
sqrt(Xyl3)	1.279	0.95	1.216	0.02494	0.01931
sqrt(Xyl5)	0.839	0.9353	0.9748	0.02242	0.01737
sqrt(G3)	2.368	1.966	2.484	0.01572	0.01218
sqrt(G4)	1.528	1.242	1.655	0.010616	0.008223
sqrt(XA3XX)	2.061	1.527	1.858	0.01229	0.00953
sqrt(XA2_3XX)	1.583	1.184	1.349	0.010451	0.008096
sqrt(XA3A3XX)	1.0187	0.6568	0.8532	0.009387	0.007271
sqrt(XA3XA3XX)	0.4647	0.3635	0.4023	0.004362	0.003379
sqrt(XA3A2_3XX)	0.7356	0.5807	0.6529	0.008442	0.006539
sqrt(XA3XA2_3XX)	1.0095	0.8237	0.9543	0.008938	0.006923
$\log_e$ (TOT-AX)	3.379	2.997	3.307	0.01194	0.00925
$\log_e$ (TOT-BG)	2.065	1.682	2.184	0.01361	0.01054
Ratio G3:G4 GOS	2.405	2.509	2.26	0.01515	0.01174
Ratio TOT-AXOS: TOT-BG	3.756	3.757	3.09	0.03691	0.02859
Sqrt (ratio M:D AXOS)	1.431	1.363	1.443	0.003936	0.003049

Table S6. p values from ANOVA of AXOS and GOS in the three types and genotypes of wheat.

Statistically significant values ($p<0.05$) are highlighted.

Some variables required transformation, square root (Sqrt) or log_e (log) to meet the assumptions of the analysis.

	Nlevel	Grain	Nlevel. Grain	Grain. genotype.Bread wheat	Grain. genotype Emmer	Grain. genotype Spelt	Nlevel.Grain. genotype. Bread	Nlevel.Grain. genotype. Emmer	Nlevel. Grain. genotype. Spelt
sqrt(Xylose)	0.62	<0.001	0.436	<0.001	0.002	<0.001	0.832	0.688	0.666
sqrt(Xyl2)	0.273	<0.001	0.698	<0.001	0.033	<0.001	0.412	0.489	0.85
sqrt(Xyl3)	0.566	<0.001	0.511	<0.001	0.004	0.547	0.08	0.906	0.464
sqrt(Xyl5)	0.359	<0.001	0.963	<0.001	<0.001	0.217	0.938	0.8	0.593
sqrt(G3)	0.001	<0.001	0.809	<0.001	0.001	0.347	0.699	0.526	0.814
sqrt(G4)	0.002	<0.001	0.5	<0.001	0.003	<0.001	0.549	0.665	0.385
sqrt(XA3XX)	0.224	<0.001	0.568	<0.001	<0.001	<0.001	0.659	0.58	0.856
sqrt(XA2_3XX)	0.411	<0.001	0.487	<0.001	0.024	0.072	0.191	0.557	0.803
sqrt(XA3A3XX)	0.07	<0.001	0.652	<0.001	<0.001	0.113	0.495	0.738	0.749
sqrt(XA3XA3XX)	0.483	<0.001	0.393	<0.001	<0.001	0.421	0.131	0.546	0.229
sqrt(XA3A2_3XX)	0.77	<0.001	0.184	0.129	0.102	0.005	0.468	0.822	0.782
sqrt(XA3XA2_3XX)	0.161	<0.001	0.492	<0.001	0.046	0.257	0.323	0.367	0.797

Table S7. Means and SEMs of AXOS in the genotypes of the three cereals grown in four environments

Some variables required transformation, square root (Sqrt) or log$_e$ (log) to meet the assumptions of the analysis.

	Bread wheat					emmer			spelt					SEM
	Akteur	Bernstein	Capo	Kometus	RGT Reform	Ramses	Roter Heidfelder	Späths Albjuwel	Attengauer	Bauländer	Comburger	Franckenkorn	Zollernspelt	
Sqrt (Xylose)	2.718	2.921	2.917	2.674	2.681	2.349	2.372	2.206	2.625	2.592	2.853	2.848	2.806	0.03628
Sqrt (Xyl2)	3.053	3.106	3.268	2.996	3.105	2.786	2.791	2.656	3.008	3.019	3.23	3.246	3.135	0.04138
Sqrt (Xyl3)	1.18	1.316	1.455	1.147	1.298	1.021	0.996	0.833	1.175	1.184	1.26	1.249	1.213	0.04319
Sqrt (Xyl5)	0.7484	0.8966	0.7627	0.9519	0.8355	0.9934	0.9995	0.813	1.0068	1.0112	0.8959	0.9712	0.9887	0.03884
Sqrt (G3)	2.232	2.382	2.63	2.28	2.315	1.949	2.044	1.906	2.504	2.465	2.517	2.445	2.487	0.02723
Sqrt (G4)	1.44	1.546	1.69	1.465	1.499	1.241	1.287	1.198	1.723	1.607	1.678	1.598	1.669	0.01839
Sqrt (XA3XX)	1.961	2.217	2.235	1.857	2.037	1.471	1.634	1.477	1.905	1.81	1.905	1.793	1.875	0.02128
Sqrt (XA2_3XX)	1.57	1.693	1.564	1.569	1.517	1.143	1.204	1.203	1.322	1.377	1.331	1.378	1.335	0.0181
Sqrt (XA3A3XX)	0.9842	1.1387	1.061	0.8568	1.0529	0.6225	0.754	0.594	0.8422	0.8394	0.8927	0.8487	0.8429	0.01626
Sqrt (XA3XA3XX)	0.4626	0.5019	0.4707	0.4215	0.4666	0.3495	0.3931	0.3479	0.4074	0.4057	0.4003	0.4079	0.3901	0.007555
Sqrt (XA3A2_3XX)	0.7447	0.7647	0.7141	0.7255	0.7289	0.5638	0.5725	0.6058	0.6115	0.6846	0.6444	0.675	0.6493	0.01462
Sqrt (XA3XA2_3XX)	0.9836	1.0782	0.9917	0.9399	1.0543	0.8011	0.8541	0.8161	0.9844	0.9412	0.9526	0.9402	0.953	0.01548

Table S8. Means and SEMs of AXOS in the genotypes of the three cereals grown in four environments with 100 and 200 kg N/Ha

Some variables required transformation, square root (Sqrt) or $\log_e$ (log) to meet the assumptions of the analysis.

	100 kg N												
	bread wheat					emmer			spelt				
	Akteur	Bernstein	Capo	Kometus	RGT Reform	Ramses	Roter Heidfelder	Späths Albjuwel	Attengauer	Bauländer	Comburger	Franckenkorn	Zollernspelt
sqrt(Xylose)	2.707	2.893	2.927	2.625	2.655	2.329	2.389	2.183	2.588	2.603	2.89	2.856	2.83
sqrt(Xyl2)	3.071	3.119	3.228	2.915	3.068	2.75	2.791	2.689	3.002	2.989	3.197	3.255	3.082
sqrt(Xyl3)	1.229	1.346	1.364	1.089	1.331	1.037	1.022	0.876	1.162	1.242	1.249	1.3	1.18
sqrt(Xyl5)	0.7492	0.919	0.7974	0.9725	0.8272	0.9905	1.0301	0.8139	0.971	0.9929	0.9305	1.0059	1.0107
sqrt(G3)	2.283	2.415	2.676	2.292	2.381	1.995	2.114	1.933	2.543	2.538	2.561	2.484	2.557
sqrt(G4)	1.47	1.549	1.71	1.468	1.539	1.267	1.319	1.207	1.742	1.656	1.713	1.606	1.72
sqrt(XA3XX)	1.981	2.212	2.235	1.834	2.05	1.503	1.639	1.481	1.912	1.834	1.919	1.792	1.904
sqrt(XA2_3XX)	1.591	1.662	1.562	1.549	1.534	1.17	1.216	1.203	1.317	1.393	1.338	1.369	1.351
sqrt(XA3A3XX)	1.0147	1.143	1.0832	0.8491	1.0625	0.6415	0.7786	0.601	0.83	0.8464	0.9102	0.8588	0.8514
sqrt(XA3XA3XX)	0.4741	0.492	0.4556	0.4164	0.4647	0.3574	0.3986	0.3447	0.408	0.4179	0.3965	0.405	0.3774
sqrt(XA3A2_3XX)	0.7703	0.767	0.71	0.715	0.7267	0.5797	0.5769	0.6107	0.591	0.684	0.6399	0.6707	0.6287
sqrt(XA3XA2_3XX)	1.0047	1.062	0.9882	0.9262	1.0685	0.8223	0.8642	0.8066	0.992	0.9573	0.9729	0.9368	0.9724

	200 kg N													SEM
	bread wheat					emmer			spelt					
	Akteur	Bernstein	Capo	Kometus	RGT Reform	Ramses	Roter Heidfelder	Späths Albjuwel	Attengauer	Bauländer	Comburger	Franckenkorn	Zollernspelt	
sqrt(Xylose)	2.73	2.949	2.907	2.724	2.706	2.37	2.355	2.229	2.662	2.582	2.816	2.839	2.782	0.05276
sqrt(Xyl2)	3.034	3.093	3.308	3.077	3.142	2.823	2.792	2.622	3.014	3.048	3.264	3.237	3.187	0.06071
sqrt(Xyl3)	1.132	1.286	1.546	1.206	1.265	1.005	0.969	0.79	1.188	1.127	1.271	1.198	1.245	0.06147
sqrt(Xyl5)	0.7476	0.8741	0.7281	0.9314	0.8437	0.9963	0.969	0.8121	1.0426	1.0294	0.8613	0.9366	0.9668	0.05498
sqrt(G3)	2.181	2.349	2.584	2.267	2.249	1.903	1.974	1.88	2.465	2.393	2.473	2.407	2.417	0.04013
sqrt(G4)	1.409	1.543	1.671	1.461	1.46	1.215	1.255	1.189	1.703	1.559	1.644	1.59	1.617	0.0264
sqrt(XA3XX)	1.941	2.221	2.236	1.879	2.023	1.439	1.629	1.472	1.899	1.786	1.891	1.793	1.845	0.03066
sqrt(XA2_3XX)	1.549	1.723	1.566	1.589	1.5	1.116	1.193	1.204	1.328	1.361	1.325	1.387	1.319	0.02533
sqrt(XA3A3XX)	0.9538	1.1346	1.0389	0.8644	1.0434	0.6035	0.729	0.5869	0.855	0.8325	0.8751	0.8386	0.8344	0.02335
sqrt(XA3XA3XX)	0.451	0.5123	0.4858	0.4265	0.4685	0.3416	0.388	0.3512	0.407	0.3936	0.404	0.4109	0.4028	0.0106
sqrt(XA3A2_3XX)	0.7192	0.7626	0.7182	0.7361	0.7311	0.5479	0.568	0.6009	0.632	0.6851	0.6488	0.6793	0.6698	0.02053
sqrt(XA3XA2_3XX)	0.9624	1.0948	0.9952	0.9535	1.0401	0.7799	0.844	0.8255	0.9772	0.9252	0.9322	0.9435	0.9336	0.02188

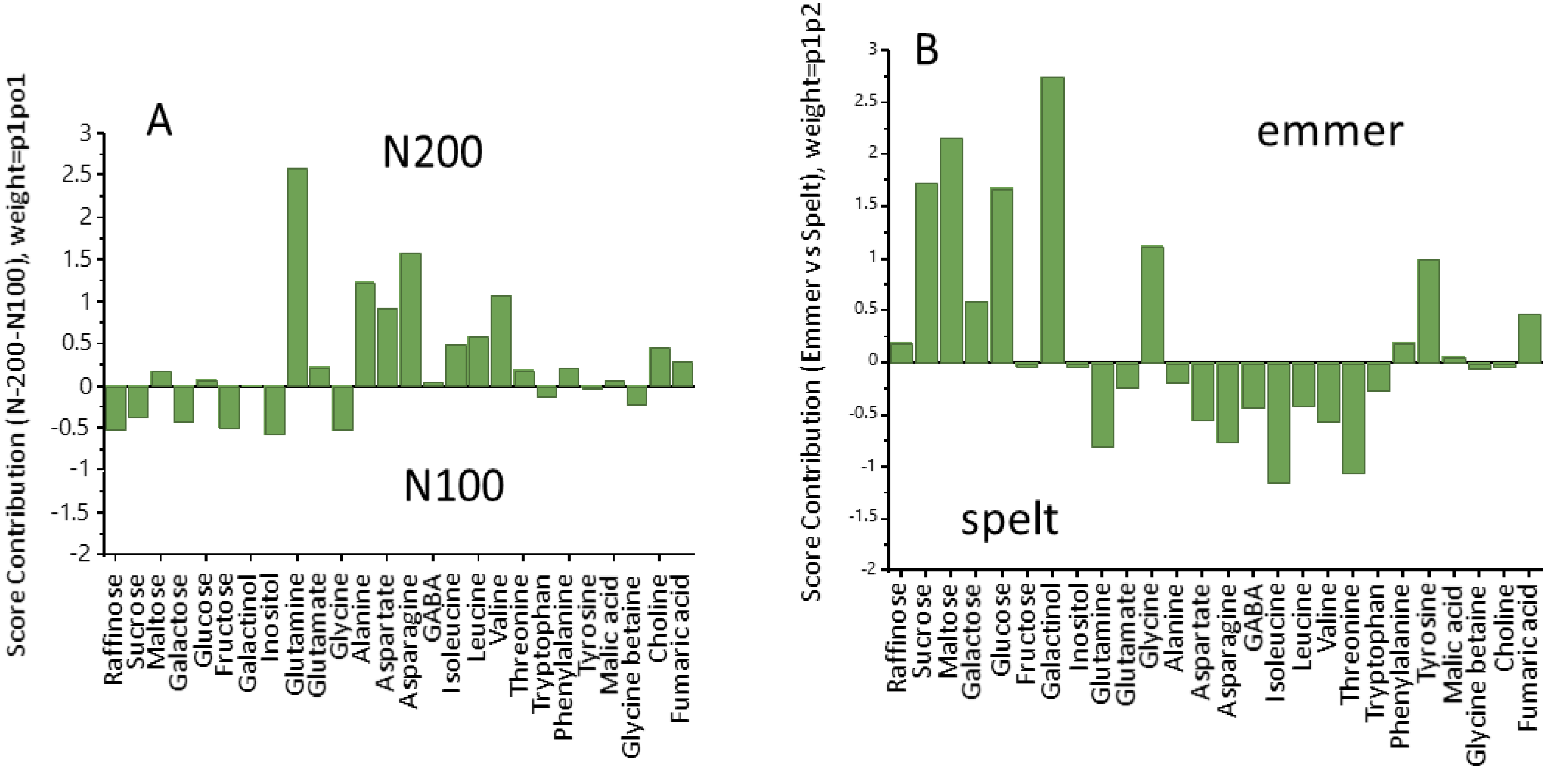
A
N200
N100
Score Contribution (N-200-N100), weight=p1po1
3
2.5
2
1.5
1
0.5
0
-0.5
-1
-1.5
-2
Raffinose
Sucrose
Maltose
Galactose
Glucose
Fructose
Galactinol
Inositol
Glutamine
Glutamate
Glycine
Alanine
Aspartate
Asparagine
GABA
Isoleucine
Leucine
Valine
Threonine
Tryptophan
Phenylalanine
Tyrosine
Malic acid
Glycine betaine
Choline
Fumaric acid
B
emmer
spelt
Score Contribution (Emmer vs Spelt), weight=p1p2
3
2.5
2
1.5
1
0.5
0
-0.5
-1
-1.5
-2
Raffinose
Sucrose
Maltose
Galactose
Glucose
Fructose
Galactinol
Inositol
Glutamine
Glutamate
Glycine
Alanine
Aspartate
Asparagine
GABA
Isoleucine
Leucine
Valine
Threonine
Tryptophan
Phenylalanine
Tyrosine
Malic acid
Glycine betaine
Choline
Fumaric acid

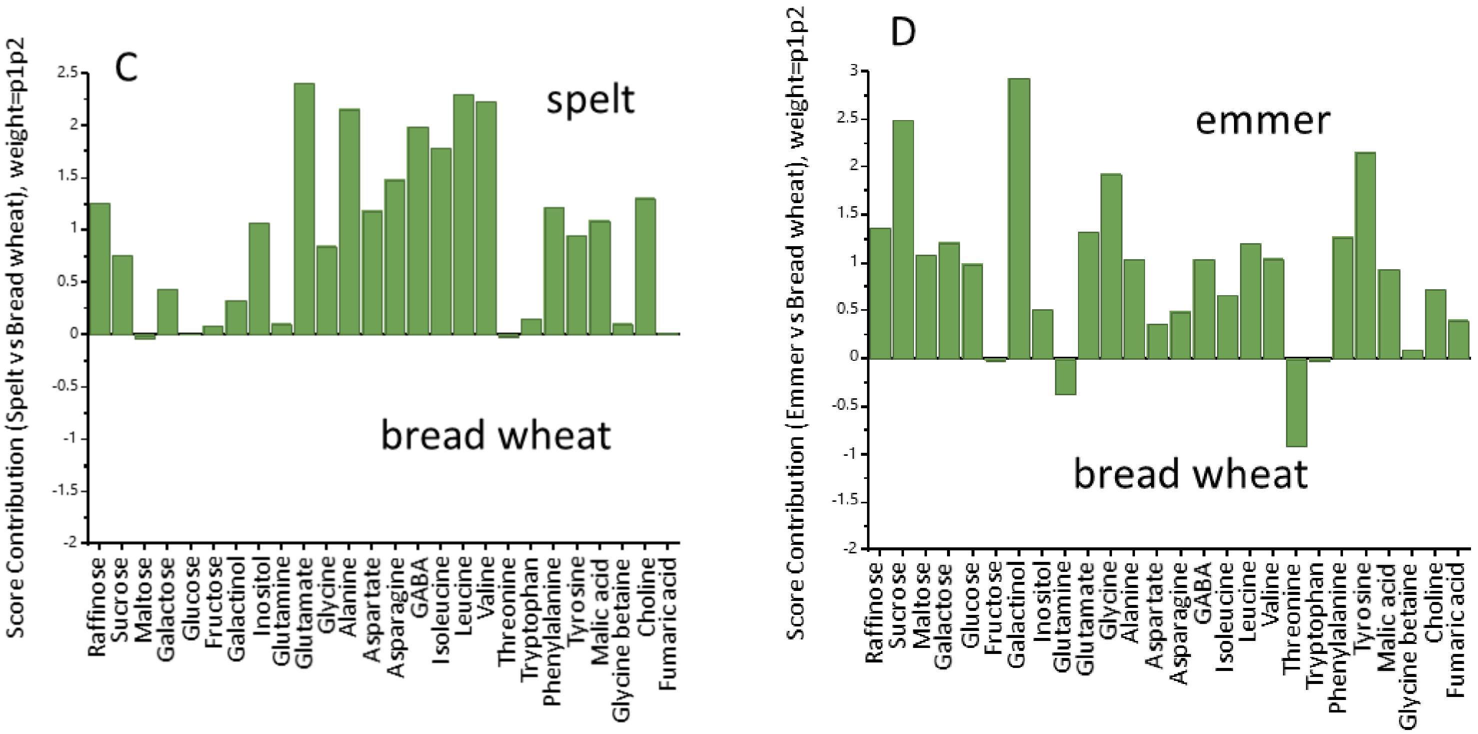

Figure S1. Difference plots showing the metabolites contributing to the separations in Figure 4B (A) and Figure 4C (B, C, D).

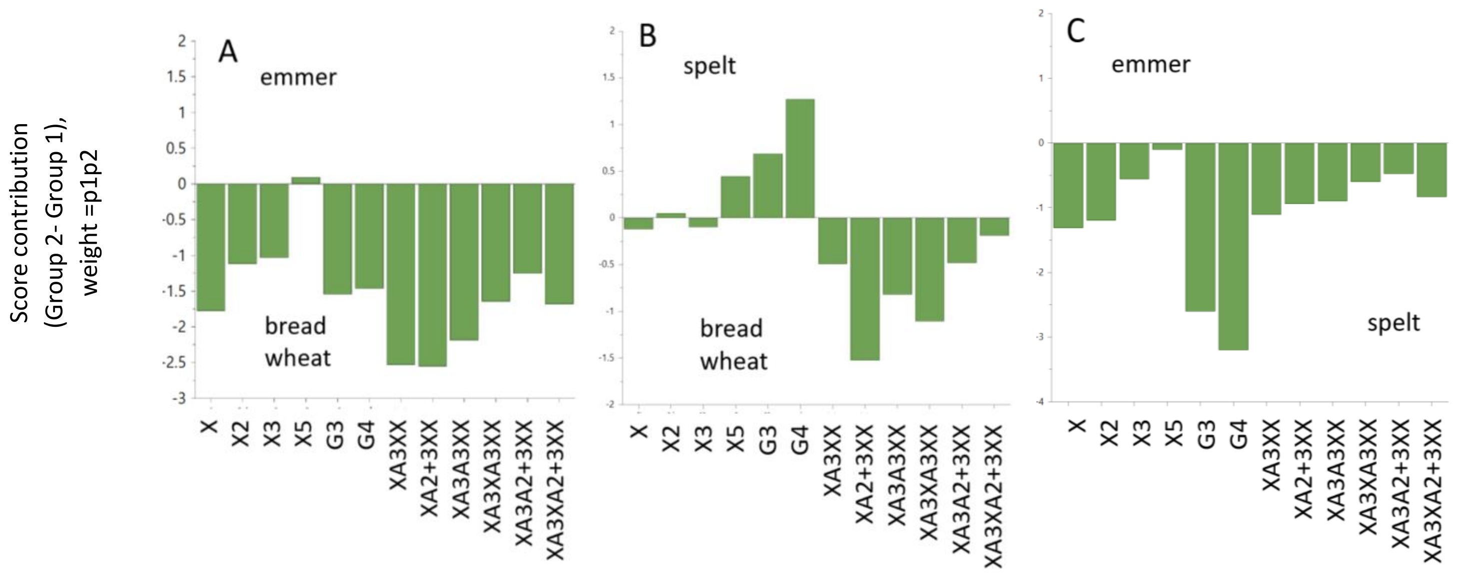

Figure S2. Difference plots showing the AXOS and GOS contributing to the separations in Figure 6A (A) and Figure 6B (B, C).
